# Corporal Punishment

**Published:** 1879-09

**Authors:** B. P. Marsh

**Affiliations:** Bloomington, Ill.


### ARTICLE XV.

### CORPORAL PUNISHMENT.

*Editors* CHICAGO MEDICAL JOURNAL AND EXAMINER:

My attention has for a number of years been called to injuries caused by occasional cases of too severe or recklessly executed punishment of school children. Foremost educators, while not believing it expedient to prohibit corporal punishment, acknowledge that injury, injustice and sometimes death even, do now and then occur from its use. These serious results may arise from fright or from concussion of the brain produced by merely jerking the child about, or—the most frequent cause—inflicting the punishment upom the child's head. I have known death to occur solely from the fright, although fatal results are not likely to arise except from immediate blows upon the head. No one form of punishment is so dangerous as boxing the child upon the ear. Not only is injury to the organ of hearing often produced, but inflammation of the brain frequently follows, and death has been the result. In the family this matter of injurious methods of punishment is not by any means beyond our influence, if we will but take pains to inform the people upon the subject. If corporal punishment is allowed at all in schools, its use ought to be carefully guarded. No teacher should be allowed to punish a child by rudely jerking it about, by striking it anywhere on the head, or with any instrument whatever, except it be flexible and with smooth edges. These requisites are best fulfilled by a rubber strap with rounded edges.

Moreover, no punishment should be permitted except it be inflicted in the presence of a principal, another teacher, or a school trustee, as a salutary check upon undue temper or excitement. Every case of corporal punishment should also be reported in writing to the board of school trustees, stating the offense of the pupil, the manner and severity of the punishment. I have known the above rules adopted by a board of school commissioners to reduce the number of cases of corporal punishment eighty-eight per cent. in one month, and the school continued meanwhile even more orderly and satisfactory than before. I am about collecting statistics of serious and fatal injuries caused by corporal punishment, and I write this article to request all the readers of the JOURNAL AND EXAMINER to forward to me statistics and history of all cases that may have come to their knowledge, with the date, place, name of child, character of punishment, and its results; also the offense for which the punishment was inflicted. Add other points, history, etc., if time and inclination permit. I urge all to give the subject the little attention needed, to write me the main facts, at least, of all cases they have known, and thereby make the report more valuable. The information thus obtained I will communicate through this journal.

B. P. MARSH, M.D.

*Bloomington, Ill.*

